# Complementary Characteristics of Correlation Patterns in Morphometric Correlation Networks of Cortical Thickness, Surface Area, and Gray Matter Volume

**DOI:** 10.1038/srep26682

**Published:** 2016-05-26

**Authors:** Jin-Ju Yang, Hunki Kwon, Jong-Min Lee

**Affiliations:** 1 Department of Biomedical Engineering, Hanyang University, Seoul, Republic of Korea

## Abstract

Morphometric correlation networks of cortical thickness, surface area, and gray matter volume have statistically different structural topology. However, there is no report directly describing their correlation patterns in view of interregional covariance. Here, we examined the characteristics of the correlation patterns in three morphometric networks of cortical thickness, surface area, and gray matter volume using a Venn diagram concept across 314 normal subjects. We found that over 60% of all nonoverlapping correlation patterns emerged with divergent unique patterns, while there were 10% of all common edges in ipsilateral and homotopic regions among the three morphometric correlation networks. It was also found that the network parameters of the three networks were different. Our findings showed that correlation patterns of the network itself can provide complementary information when compared with network properties. We demonstrate that morphometric correlation networks of distinct structural phenotypes have different correlation patterns and different network properties. This finding implies that the topology of each morphometric correlation network may reflect different aspects of each morphometric descriptor.

Morphometric-based correlation networks (MCNs) have been commonly constructed using divergent structural phenotypes such as cortical thickness, cortical surface area, and gray matter (GM) volume to investigate the patterns of large-scale structural association between brain regions in healthy subjects and diverse patients such as those with Alzheimer’s disease, multiple sclerosis, schizophrenia, and epilepsy (see Supplementary Table S1). While most studies to date have employed MCNs without explicit distinction of structural phenotypes, some studies have begun to look at the difference in the network topology between the structural phenotypes. Sanabria-Diaz *et al.* found that the topologies of cortical thickness and surface area networks were significantly different, demonstrating that both capture distinct properties of the interaction or different aspects of the same interaction (e.g., mechanical, anatomical, or chemical) between brain structures^1^. Anand *et al.* also evaluated a comparison of MCN properties with surface area, GM volume, and thickness on the same population to show that the topology of cortical thickness, surface area, and volume networks was significantly different^2^.

While these two studies evaluated the network parameters that summarize an entire topology down to a single number such as clustering coefficient, characteristic path length, global network efficiency, and small-worldness, the correlation pattern of the network itself could provide a complementary insight to compare the topology of different structural phenotypes. The network parameters provide a global measure for an entire topology and local measures allowing the edge properties for a node, and are thus attractive because of their simplicity; however, they can only compare the networks statistically. By contrast, the correlation patterns of the networks reveal the spatial distribution of the edges and can detect the edge-based similarity of topological features inherent in each network. For example, Gong *et al.* found that approximately 35–40% of cortical thickness correlations showed convergent diffusion connectivity across the cerebral cortex^3^. Although MCNs have the same number of nodes with the same regions, they may have different interregional correlation patterns driven by distinct cellular mechanisms as the result of a distinct genetic origin. Different genetic factors influence patterns of structural covariance most strongly within different brain networks^4^,^5^,^6^,^7^,^8^; that is, there seem to be network-specific genetic influences^9^. Chen *et al.* showed that the differences in genetic influence on cortical surface area were along an anterior–posterior axis; the differences in genetic influences on cortical thickness were along a dorsal–ventral axis in the same cohort^8^,^10^. The distinct structural phenotypes could result in different interregional correlation patterns, which in turn have an effect on the network properties.

To our knowledge, the quantitative evaluation of the similarity of the correlation patterns between the network topology of distinct structural phenotypes including cortical thickness, surface area, and GM volume has not been considered in detail. The similarity of the correlation patterns can be evaluated by the overlap ratio (OR) of the spatially distributed edges and the types of edge. A Venn diagram concept dividing all possible cases of the edges is useful to investigate the OR of the spatially distributed edges in a detailed manner: common edges overlapping in all three networks, three parts of overlapping edges in two networks only, and three parts of nonoverlapping edges in each network exclusively (Fig. 1). Furthermore, the classification of edge types according to inter- and intrahemispheric connections may provide useful information regarding the edge patterns in terms of lateralization: (1) homotopic connection to indicate the same areas in opposite hemispheres; (2) the left and right ipsilateral connection to indicate different areas in the same hemispheres; and (3) heterotopic connection to indicate different areas in opposite hemispheres^11^. Therefore, the number of edges belonging to each connection type in seven partitions respectively yields quantitative information about the correlation patterns of MCNs.

In this study, we partitioned the spatially distributed edges into seven parts and classified them into four different edge types to characterize the various correlation patterns of cortical volume, thickness, and surface area networks. We also calculated network parameters to explore whether the characteristics of correlation pattern have an effect on the network parameters.

## Results

### Similarity of the correlation pattern

The overlap ratio (OR) is the number of pairs present in each partition divided by the number of all edges surviving the threshold in the selected network sparsity across 314 normal subjects from the principal data set. *V*_*TAV*_ is the set of the common edges of the three networks; *V*_*TA*_ is the set of the shared edges of thickness and surface area networks; *V*_*TV*_ is the set of the shared edges of thickness and GM volume networks; *V*_*AV*_ is the set of the shared edges of surface area and GM volume networks; *V*_*TO*_ is the set of the exclusive edges in thickness networks; *V*_*AO*_ is the set of the exclusive edges in surface area networks; and *V*_*VO*_ is the set of the exclusive edges in GM volume networks. We calculated the OR in each partition and plotted the ORs according to the sparsity range of 8–24%, where *OR*(*V*_*TAV*_) was 10–12%, *OR*(*V*_*TA*_), *OR*(*V*_*TV*_) and *OR*(*V*_*AV*_) were under 10%, and *OR*(*V*_*TO*_), *OR*(*V*_*AO*_) and *OR*(*V*_*VO*_) were 20–25% (Fig. 2). The results indicate that each network had different correlation patterns that were distinct from each other. The observed *OR*(*V*_*TAV*_), *OR*(*V*_*TV*_), *OR*(*V*_*TV*_) and *OR*(*V*_*AV*_) were higher, and the observed *OR*(*V*_*TO*_), *OR*(*V*_*AO*_) and *OR*(*V*_*VO*_) were lower than the expected value from 1000 simulated pairs of random networks with statistical significance under *P* < 0.05.

### Edge types in each partition

We examined the edge types and the signs in each partition to detect the characteristics of spatially distributed edges. The numbers of edges of four types were counted and divided by the total number of edges in each partition along the entire sparsity range (Fig. 3). The edges of *V*_*TAV*_ were mostly shown ipsilateral with slightly more left-dominant lateralized connections than right connections and bilateral homotopic connections. The correlation patterns of *V*_*TA*_ and *V*_*AV*_ were stronger in order of ipsilateral (R > L) connections, and the correlation patterns of *V*_*TV*_ showed a different order of high heterotopic connections. The correlation patterns in *V*_*TO*_, *V*_*AO*_, and *V*_*VO*_ were considerably different from each other. The correlation patterns of *V*_*TO*_ and *V*_*VO*_ showed highly heterotopic connections, and the correlation patterns of *V*_*AO*_ were in order of right-dominant ipsilateral connections. Furthermore, only positive associations were shown in *V*_*VO*_, whereas both positive and negative associations were revealed in *V*_*TO*_ with heterotopic connections and *V*_*AO*_ with mostly ipsilateral connections.

### Hub regions

We examined hub regions of *M*_*T*_, *M*_*A*_, and *M*_*V*_ and in each partition of *V*_*TAV*_, *V*_*TA*_, *V*_*TV*_, *V*_*AV*_, *V*_*TO*_, *V*_*AO*_, and *V*_*VO*_ (Fig. 4). We found that the detected hub regions of *M*_*T*_ had the maximum Jaccard index with *V*_*TO*_ (Jaccard index: 0.53), and those of *M*_*V*_ with *V*_*VO*_ (Jaccard index: 0.6). Note that the detected hub regions of *M*_*A*_ had a maximum Jaccard index in *V*_*AV*_ (Jaccard index: 0.5) and the second in *V*_*AO*_ (Jaccard index: 0.39) (Table 1).

### Topological properties of the MCNs

We compared the global network parameters including clustering coefficient, characteristic path length, small-worldness index, and global efficiency according to the sparsity range of 8–24% (see Supplementary Fig. S7). Three MCNs showed small-world network properties. Furthermore, three MCNs showed statistically different network parameters from ANOVA test with *P* < 0.05, and significantly reduced clustering coefficient, characteristic path length, and increased global efficiency in common. The thickness network showed higher clustering coefficient than those of the surface area and GM volume networks.

### Subgroup analysis

Group differences were also investigated by comparing the ORs (1) between male and female, (2) young and old, (3) patients with dementia and old normal controls. The ORs of each group were similar to those of the principal data, for example, over 60% of all nonoverlapping edges with divergent patterns, approximately 10% of all common edges with the edges showing ipsilateral and homotopic pairs. There were little differences between both comparisons in the ORs (*P* < 0.05) (see Supplementary Fig. S1). The edge types of negative correlation in *V*_*VO*_ were shown in patients with dementia and old group (see Supplementary Fig. S2). The hub regions of MCNs were highly overlapped to those of each partition in each group, with results shown in Supplementary Fig. S3 and Supplementary Table S41.

### Replication of the study

In order to demonstrate the reproducibility of the results with larger sample size and with more young adult subjects, we calculated the ORs, edge types and hub regions. To investigate the group difference, we subdivided the total subjects of the replication data into males and females and analyzed them separately, with results shown in Supplementary Figs S4–S6. As shown in Supplementary Fig. S4, overall patterns of the ORs were similar to those of the principal data, although the ORs of *V*_*TAV*_ were higher than those of the principal data. There were some different patterns showing the edge types of negative correlation in total and male group while the edge types of negative correlation in female group was similar to those of the principal data (see Supplementary Fig. S5). The hub regions of MCNs were similar to those of each partition in each group (see Supplementary Fig. S6, and Supplementary Table S5).

## Discussion

In this study, we examined the characteristics of correlation patterns to determine the difference in structural topology between the three MCNs of cortical thickness, surface area, and GM volume. This may allow complementary accounting for spatial and topological information when combined with network parameters.

Our findings showed that over 60% of all nonoverlapping correlation patterns emerged from distinct structural phenotypes, that is, thickness, surface area, and GM volume-based networks. The findings support the hypothesis that the different structural phenotypes of cerebral cortex have different network properties^1^,^2^. Sanabria-Diaz *et al.* and Anand *et al.* demonstrated the hypothesis statistically comparing the network parameters such as clustering coefficient, characteristic path length, and global efficiency. The network parameters may help identify significant differences in the network topology. While the global network parameters gave us an abstraction of the complex human brain topology as a single value, we provided the complementary characteristics of network topology such as the percentage of similarities between MCNs and the types of edges. We can better understand and explain quantitatively the differences of brain network topology in terms of how they are similar or dissimilar between MCNs and which types of edge they have. In this study, we observed that the *OR*(*V*_*TO*_), *OR*(*V*_*AO*_) and *OR*(*V*_*VO*_) was respectively 20% with the different correlation patterns in case of the only existing edges in each network. The correlation patterns were considerably different than those of *V*_*TO*_ and *V*_*VO*_, which had more heterotopic connections, while the correlation patterns of *V*_*AO*_ showed right-dominant ipsilateral connections (R > L). We found only positive associations were shown in *V*_*VO*_, whereas both positive and negative associations were revealed in *V*_*TO*_ and *V*_*AO*_. The negative correlation patterns of *V*_*TO*_ were shown to be the pairs of regions between the frontal cortex (i.e. superior frontal gyrus) and the temporal cortex (i.e. parahippocampal gyrus), and those of *V*_*AO*_ existed predominantly in the pairs of regions between the frontal cortex (i.e. superior frontal gyrus (dorsolateral, medial), supplementary motor area) and parieto-occipital cortex. The findings may be related to the distinct genetic mechanisms supporting the findings of previous studies^8^,^10^. Chen *et al.* reported the dorsal–ventral (D-V) regions, such as the motor/premotor, parietal, and occipital region (the dorsal cluster) from prefrontal and temporal regions (the ventral cluster) division as the most distinctive partition in the genetic patterns of cortical thickness. The anterior–posterior (A-P) regions, such as the motor and sensory cortices, like the basic frontal/nonfrontal division, were observed in the genetic patterning of the cortical surface area^8^,^10^. It is well known that cortical thickness and surface area are influenced by distinct cellular mechanisms because of their distinct genetic origins^1^,^6^,^12^,^13^,^14^,^15^. The measures of cortical surface area and cortical thickness represent respectively the number of cortical columns as an overall degree of folding and the amount of cell density in the cortex^16^, although the measure of cortical volume captures some combination of both cortical thickness and surface area such as structural phenotype and genetic factors, because the volume is calculated by multiplying cortical thickness and surface area. However, the exact biological nature of this relationship remains unknown and warrants further study.

The correlation patterns of two-by-two matrices such as *V*_*TA*_, *V*_*TV*_, and *V*_*AV*_ showed quite different patterns between them. The differences could be related to intrinsic characteristics or different evolutionary development, or genetic effects of each morphometric descriptor. The interactions between the two-by-two networks may reflect a complex mechanism of structural covariance. Currently, it is difficult to compare our findings with previous work. It is important to investigate the interaction between the morphometric descriptors as to how those interactions are related to the information about the complex mechanical, genetic, and chemical relationships between different brain sites^1^. The exact biological nature of these relationships may be revealed by future study.

We found an OR of approximately 10% between the MCNs with all positive correlations. It is also important to note that the correlation patterns of *V*_*TAV*_ showed left-dominant ipsilateral (L > R) and bilateral homotopic connections, which was consistent with previous findings by Gong *et al.* who reported that 30–40% of only positive thickness correlations shared white matter connectivity in ipsilateral or bilateral homotopic regions^3^. Lerch *et al.* were the first to show that cortical thickness correlations qualitatively match a diffusion tensor imaging traced track, implying that anatomical connectivity could be measured indirectly using information from the cortical surface^17^. Our findings are consistent with the hypothesis of anatomical connectivity induced by mutually tropic influences^1^,^3^,^11^,^15^,^17^,^18^,^19^,^20^ supporting the common morphometric changes of three structural phenotypes in the same direction leading to positive correlations between related regions.

We examined which hub regions of MCNs were similar to those of each partition. The hub regions of the cortical thickness network were closer to that of *V*_*TO*_. The hub regions of a surface area network were likely to be one of *V*_*AV*_ and *V*_*AO*_. The hub regions of GM volume networks were similar to that of *V*_*VO*_. The similarity between hub regions of MCN networks and subnetworks of each partition showed that the hubs were strongly associated with their intrinsic correlation patterns. The hub has an important role in integrating and distributing information because of the number and position of its correlations in a network. We also examined the network parameters such as the clustering coefficient, characteristic path length, and global efficiency; these were significantly different between MCNs, which was consistent with previous studies^1^,^2^.

We investigated the group differences of the characteristics of correlation patterns between male and female, young and old, patients with dementia and old normal controls from the principal data set. We found that all subgroups had similar characteristics of correlation patterns in general, whereas patients with dementia and old normal subjects had the edges of the negative correlation in *V*_*VO*_. It might reflect altered inter-regional correlations in aging^21^. However, the underlying biological nature of the morphological correlations is still unknown.

We demonstrated that the reproducibility of the results were similar with the characteristics of correlation pattern showing over 60% of all nonoverlapping correlation patterns, 10–18% of all common edges with ipsilateral and homotopic pairs with only positive correlations. We examined some different types of negative correlation in total and male group, but overall trend of negative correlations were shown in bilateral heterotopic regions while those of positive correlations are mostly among ipsilateral or homotopic regions supporting with previous study^3^. It is still unknown the existence of different mechanisms of the positive and negative correlation of morphology.

This study has several limitations. The main limitation is that, we used the AAL atlas for a node definition, as can be done by alternative choices of different brain parcellations such as high-resolution parcellations or another anatomical atlas like Brodmman, but provides biological and anatomical meaningful cortical regions comparing to high-resolution parcellations^23^. Since previous studies have successfully employed this parcellation scheme, we used AAL atlas to keep our findings consistent with the previous studies^1^,^3^,^21^. It is also notable that inter-regional correlation measure was evaluated by Pearson correlation test^3^,^15^,^21^. Adoption of different methods such as partial correlation or mutual information may have some effect on the pattern of results^2^. The different connectivity measures need to be addressed in the future. Furthermore, although we tried to remove the confounding effect by using the regression model, it might not explicitly deal with still remain. Another consideration is that, because cortical thickness and surface area were defined on the cerebral cortex, we excluded the sub-cortical structures. And we constructed the binary adjacency matrix because of its simplicity for interpretation. It is needed to address in terms of weighted network.

Our results demonstrated that morphometric correlation networks of distinct structural phenotypes have different correlation patterns and different network properties.

## Methods

### Data acquisition

We conducted the experiments using two data sets in this study: (1) the set of cross-sectional data from the Open Access Series of Imaging Studies (OASIS) database (www.oasis-brains.org), referred to as the principal data set of the study and (2) Human Connectome Project (HCP) data (www.humanconnectome.org), referred to as the replication data set. Supplementary Data provides more information about study population and image acquisition.

### Image preprocessing for a morphometric descriptor

We computed three cortical features such as cortical thickness, surface area and GM volume. CIVET software was used to extract two cortical surfaces, the inner and outer surfaces, which are composed of 3-D meshes. The procedures are well validated and have been extensively described^23^,^24^,^25^,^26^,^27^. Further image processing was described in Supplementary Methods section. Cortical thickness was calculated as the Euclidean distance between the corresponding vertices of inner and outer surfaces using a t-link method^23^. Surface area was measured by calculating the Voronoi area assigned to any vertices^28^. Cortical thickness and surface area were blurred using a 20 mm full-width half-maximum surface-based diffusion kernel^29^,^30^. GM volume was obtained from the segmented GM images in native space.

### Morphometric network construction

We divided the cortical surface into 78 regions (39 for each hemisphere) to identify nodes of the MCN with the automated anatomical labeling (AAL) template excluding the cerebellum and subcortical regions (i.e. amygdala, caudate, hippocampus, pallidum, putamen, and thalamus). We applied the same procedures described in the previous work to cortical parcellation^3^,^22^ and network construction^3^. Supplementary Methods section provides more details of the network construction. Finally, three 78 × 78 symmetric correlation matrices were obtained for cortical thickness, cortical surface area, and GM volume. A binary adjacency matrix with 1 or 0 indicating the simple presence or absence of an edge was created by thresholding the correlation matrix in terms of sparsity^3^. Sparsity measures the percentage of the number of existing edges in all possible links^31^. Because there is no definitive criterion for a single threshold, we observed the results through a sparsity threshold of 8–24% satisfying a fully connected MCN ensuring the same number of suprathreshold regional pairs for all the networks^3^. In equations (1, 2, 3) below, *T*, *A*, and *V* indicate the adjacency matrix of cortical thickness, surface area, and GM volume, respectively, and *M*_*T*_, *M*_*A*_, and *M*_*V*_ are the set of the elements representing edges in each adjacency matrix:


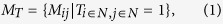



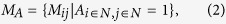



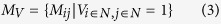


where index *i* and *j* indicates the node i^th^ row and j^th^ column in an adjacency matrix and *N* is the set of all nodes in the networks. Note that only the elements of upper triangles in the adjacency matrix were considered because the adjacency matrix is symmetric.

### Partitioning the edges

**Figure 1:**
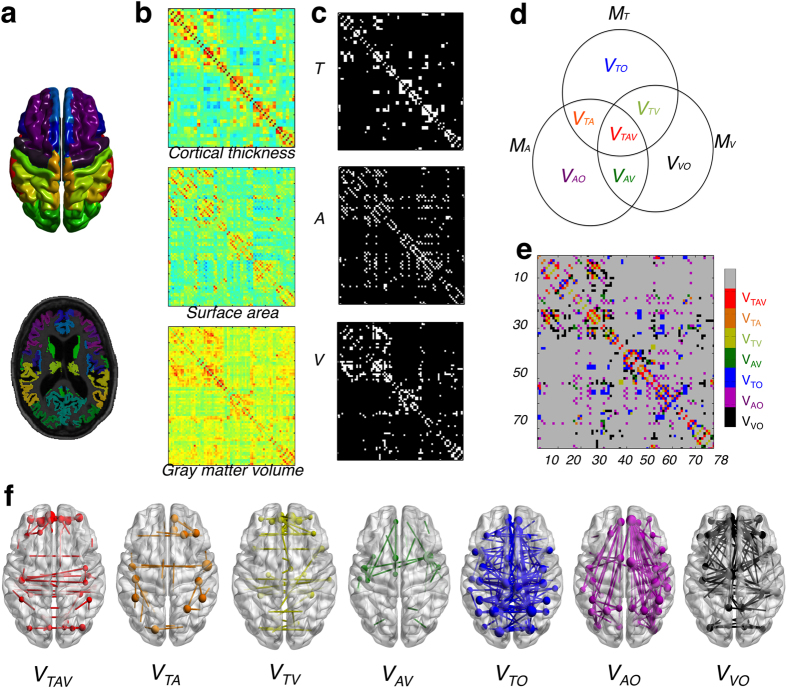
Schematic showing processing for constructing morphometric correlation networks (MCN) and the Venn diagram concept. (**a**) The AAL template masks on the cortical surface and volume on the MNI 152 template. Each color represents 78 cortical regions. (**b**) Interregional correlation matrix in each cortical thickness, surface area, and gray matter (GM) volume was performed using Pearson correlation analysis from the principal data set, after removing the age, sex, and global measures. The colors of the matrix ranged from − 1 to 1. (**c**) The adjacency matrix was thresholded at 8% of sparsity. *T*, *A*, and *V* indicate the adjacency matrix of cortical thickness, surface area, and GM volume, respectively. (**d**) A Venn diagram concept was employed to identify edges of all possible cases in three networks. *M*_*T*_, *M*_*A*_, and *M*_*V*_ are the set of the elements representing edges in each adjacency matrix. *V*_*TAV*_, *V*_*TA*_, *V*_*TV*_, *V*_*AV*_, *V*_*TO*_, *V*_*AO*_, and *V*_*VO*_ were the set of the elements representing edges in each partition. (**e**) The colors of the element in the matrix indicate the edges in each partition. (**f**) The regional pairs in each partition for visualization (8% sparsity).

**Figure 2:**
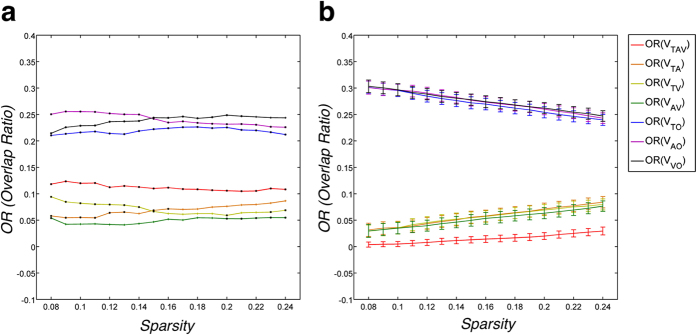
The overlap ratio (OR) in seven partitions. (**a**) The plot figure shows the observed OR of each partition as a function. Data points with black color indicate statistically significance (*P* < 0.05). The significance was reached if less than 5 percentile in the ORs distribution of the random networks. (**b**) The ORs of 1000 random simulated networks show the mean and standard deviation of each partition as a function.

**Figure 3:**
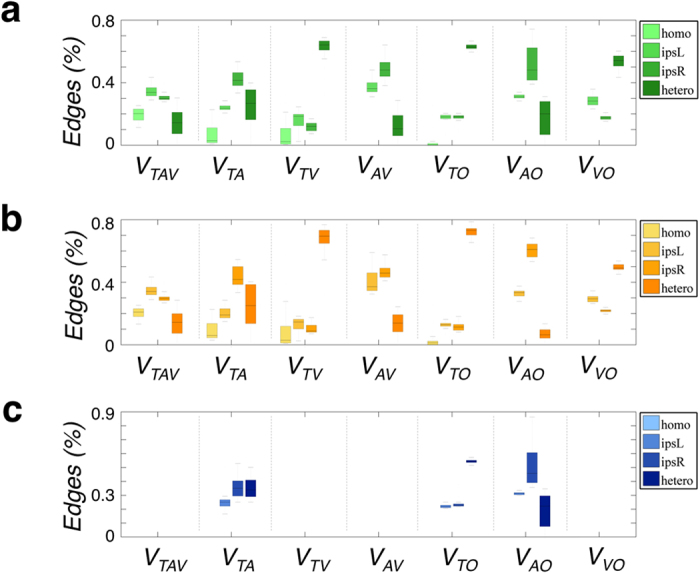
The percentages of the four types of edges, that is, the homotopic, the left and right ipsilateral, and heterotopic connections were plotted. (**a**) The edges show both signs, with positive and negative correlation coefficients between regions. (**b**) The edges show only positive correlation. (**c**) The edges show only negative correlation. In each box, the central mark is the median, the edges of the box are the 25^th^ and 75^th^ percentiles, and the box color indicates the connection type.

**Figure 4:**
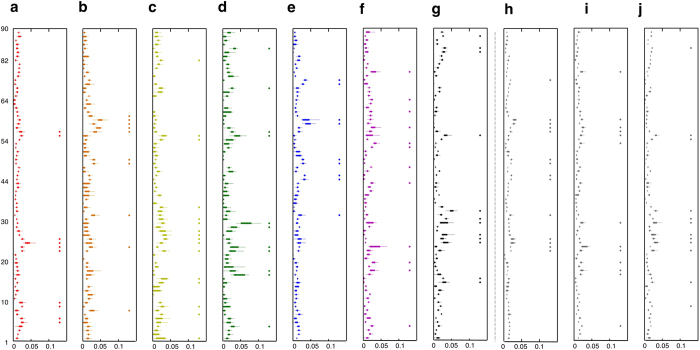
The hub regions in seven partitions and MCNs. Each partition (**a**–**j**) was marked as follows: (**a**) *V*_*TAV*_, (**b**) *V*_*TA*_, (**c**) *V*_*TV*_, (**d**) *V*_*AV*_, (**e**) *V*_*TO*_, (**f**) *V*_*AO*_, (**g**) *V*_*VO*_, and (**h**) *M*_*T*_, (**i**) *M*_*A*_, and (**j**) *M*_*V*_ indicate the adjacency matrix of cortical thickness, surface area, and gray matter volume, respectively. In each box, the central mark is the median, the edges of the box are the 25^th^ and 75^th^ percentiles of the normalized degree of a node (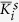) at each sparsity. *H*_*i*_ is the average of the normalized degree of a node (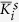) over the sparsity range. The hub regions were defined as the nodes with higher than mean (*H*_*i*_) + standard deviation (*H*_*i*_) and marked as circle with same color in each box plot. Abbreviations for the cortical regions and number of AAL were listed in Supplementary Table S3.

All the edges in the three MCNs were partitioned into seven parts: portions of their common edges overlapped in three networks, three parts of overlapped edges between two networks, and three parts of exclusive edges in each network (Fig. 1). Each partition was defined as follows.


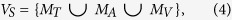



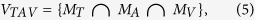



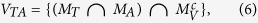



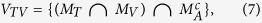



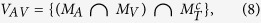



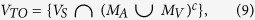



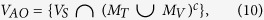



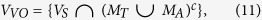


*V*_*S*_ is the set of the union edges of the three networks. *V*_*TAV*_, *V*_*TA*_, *V*_*TV*_, *V*_*AV*,_ *V*_*TO*_, *V*_*AO*_ and *V*_*VO*_ were the set of the elements representing edges in each partition.

### Identifying characteristics of correlation patterns

We defined the OR of the spatially distributed edges as follows.


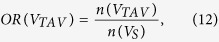



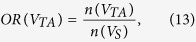



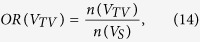



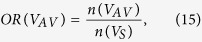



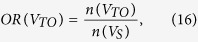



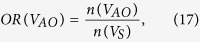



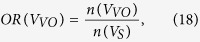


Note that the total sum of ORs is 1. We classified the edges in each partition into the four types to investigate the connection patterns in terms of lateralization: (1) homotopic connection between the same areas in opposite hemispheres; (2) the left and (3) right ipsilateral connection between different areas in the same hemispheres; and (4) heterotopic connection between different areas in opposite hemispheres^11^. Note that we have separated the ipsilateral connections into left and right hemispheres, thereby expanding the three types described by Mechelli *et al.*^11^. The edges of each connection type in each partition at each sparsity level were counted, and the number of edges of each case was divided by the total number of edges in the corresponding partition to normalize the value. We also examined the positive and negative correlation patterns between brain regions.

### Identifying hub nodes

We calculated the network parameters such as nodal degree, clustering coefficient, characteristic path length, and global efficiency, which represent the topological properties of the network, to elucidate the associations between the correlation patterns and the network parameters. The definitions and formulations of the network parameters were described in Supplementary Table S2.

Hubs can be defined as the nodes with either high degree or high centrality^31^,^32^. The degree of a node (*k*_*i*_) indicates the number of links connected to a node. In this study, the degree of a node was divided by degree sum of all nodes at each level of sparsity to normalize the value as follows.


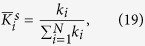


_The average of the normalized degree of a node over the sparsity range is defined as:_


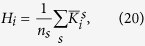


where *n*_*s*_ is the number of the sparsity range from 8–24% with 1% increment, *s* = {*0.08:0.01:0.24*}.

Nodes with the average of the normalized degree of a node (*H*_*i*_) larger than the mean plus standard deviation are considered as hubs of a network. We calculated the network parameters using the Brain Connectivity Toolbox (www.brain-connectivity-toolbox.net) and used BrainNet Viewer for visualization of networks on brain templates (www.nitrc.org/projects/bnv).

### Statistical analysis

A non-parametric test was employed to assess the statistical differences of ORs between observed and simulated pairs of random networks. First, matched random networks with all morphological measures were constructed with the same node degree of distribution^18^. Then, we recomputed the partitioning edges and ORs. This procedure was repeated 1000 times for each sparsity threshold and significance was reached if less than 5 percentile in the ORs distribution of the random networks.

We measured the Jaccard index of the hub regions of *M*_*T*_, *M*_*A*_, and *M*_*V*_ and in each partition of *V*_*TAV*_, *V*_*TA*_, *V*_*TV*_, *V*_*AV*_, *V*_*TO*_, *V*_*AO*_, and *V*_*VO*_ to see the overlap of the hub regions quantitatively. If *A* and *B* are the hub node sets from two networks, then the Jaccard index of the hub regions is defined as:


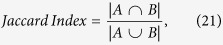


where 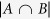 is the number of hub nodes that overlap in network *A* and *B*, and 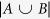 is the total number of hub nodes in the two networks. Note that the Jaccard index, ranging from 0 (no overlap) to 1 (perfect overlap), indicates how much overlap there is between the hub sets *A* and *B*.

We also obtained bootstrap samples by selecting random samples with replacement from principal data (n = 251, 80% of 314 samples)^2^,^3^. MCNs were constructed and network parameters were computed from the bootstrap samples. This procedure was repeated 1000 times and analysis of variance (ANOVA) test was performed to compare the network parameters among the three MCNs.

To evaluate the difference in ORs between groups; (1) male and female, (2) young and old, (3) patients with dementia and old, we used a permutation test. Subgroups were randomly reassigned to one of two groups comprising the same number of subjects. MCNs were then reconstructed, the ORs were calculated at each sparsity range, and the between group difference in ORs was calculated for each permutation. This procedure was repeated 1000 times. The p value was then calculated as the proportion of entries in the corresponding permutation distribution that were greater than the observed group difference.

### Replication analysis

To test the reproducibility of the results, we performed the same analyzing procedures aforementioned to an independent dataset of HCP data, which is composed of young adult subjects (age ranged from 20–35 years old).

## Additional Information

**How to cite this article**: Yang, J.-J. *et al.* Complementary Characteristics of Correlation Patterns in Morphometric Correlation Networks of Cortical Thickness, Surface Area, and Gray Matter Volume. *Sci. Rep.* **6**, 26682; doi: 10.1038/srep26682 (2016).

## Supplementary Material

Supplementary Information

## Figures and Tables

**Table 1:** Jaccard index for the coincident hub nodes.

	V_TAV_	V_TA_	V_TV_	V_AV_	V_TO_	V_AO_	V_VO_
M_T_	0.263	0.500	0.111	0.136	0.533*	0.083	0.083
M_A_	0.211	0.211	0.115	0.500*	0.048	0.389	0.087
M_V_	0.294	0	0.556	0.211	0	0	0.600*

The Jaccard index was calculated for the coincident hub regions between MCNs of *M*_*T*_, *M*_*A*_ and *M*_*V*_ and subnetworks of each partition such as *V*_*TAV*_, *V*_*TA*_, *V*_*TV*_, *V*_*AV*_, *V*_*TO*_, *V*_*AO*_, *V*_*VO*_. The hub regions of each network are marked in Fig. 4. Asterisk indicates the highest Jaccard index between hub regions of MCNs and subnetworks of each partition.
