# Supplementary material for: Complementary Characteristics of Correlation Patterns in Morphometric Correlation Networks of Cortical Thickness, Surface Area, and Gray Matter Volume
Source: Sci Rep. 2016 May 26;6:26682. doi: 10.1038/srep26682 (PMC4881013; doi:10.1038/srep26682)
Supplement: Supplementary Information [file srep26682-s1.pdf]

# Complementary Characteristics of Correlation Patterns in Morphometric Correlation Networks of Cortical Thickness, Surface Area, and Gray Matter Volume

Jin-Ju Yang, Hunki Kwon, Jong-Min Lee *

Department of Biomedical Engineering, Hanyang University, Korea

***Corresponding author:**

Jong-Min Lee, PhD

Dept. of Biomedical Engineering, Hanyang University

Mailing Address: 319 Sanhak-kisulkwan

222 Wangsipri-ro, Sungdong-gu, Seoul, KOREA, 133-791

Office: +82-2-2220-0685, Lab: +82-2-2220-0697, FAX: +82-2-2296-5943

E-mail: ljm@hanyang.ac.kr

**Supplementary Table S1-S5,**

**Supplementary Fig. S1-S7,**

**Supplementary Data and Methods**

**Supplementary Table S1** Previous studies using morphometric-based correlation networks (MCNs) with various structural phenotypes.

| Previous Studies | Structural Phenotypes | Data |
|---|---|---|
| [1]He, Chen et al. 2007 | Cortical thickness | HS |
| [2]Bassett, Bullmore et al. 2008 | GM volume | Schizophrenia |
| [3]Chen, He et al. 2008 | Cortical thickness | HS |
| [4]He, Chen et al. 2008 | Cortical thickness | AD |
| [5]He, Dagher et al. 2009 | Cortical thickness | MS |
| [6]Lv, Li et al. 2010 | Cortical thickness | HS |
| [7]Raj, Mueller et al. 2010 | Cortical thickness | Epilepsy |
| [8]Sanabria-Diaz et al. 2010 | Cortical thickness, surface area | HS |
| [9]Bernhardt, Chen et al. 2011 | Cortical thickness | Epilepsy |
| [10]Fan, Shi et al. 2011 | GM volume | Pediatric |
| [11]Zhou, Wang et al. 2011 | GM, WM, CSF volume | AD, MCI |
| [12]Hosseini, Koovakkattu et al. 2012 | GM volume | Breast cancer |
| [13]Shi, Yap et al. 2012 | GM volume | Neonates |
| [14]Wu, Taki et al. 2012 | GM volume | HS |
| [15]Hosseini, Black et al. 2013 | Cortical thickness, surface area | Dyslexia |

GM, gray matter; WM, white matter; CSF, cerebrospinal fluid; HS, Healthy subjects; AD, Alzheimer's disease; MCI, mild cognitive impairment; MS, Multiple sclerosis.

**Supplementary Table S2** Definitions and formulations of network parameters used in this study. The detailed definitions and formulations were described in a previous study[16].

| Parameter | Definitions | Formulations |
|---|---|---|
| Degree | The degree of a node ($k$) indicating the number of links connected to a node | $k_i = \sum_{j \in N} a_{ij}$ <br> where $N$ is the set of all nodes in the networks and $a_{ij}$ is a link (i.e. edge) from a node $i$ to a node $j$. |
| Clustering Coefficients | The clustering coefficient ($C$) measuring the whole brain segregation | $C = \frac{1}{n}\sum_{i \in N} C_i = \frac{1}{n}\sum_{i \in N} \frac{2t_i}{k_i(k_i - 1)}$ <br> where $C_i$ is a clustering coefficient of node ($C_i = 0$, for $k_i < 2$), $t_i$ is the number of triangles around a node $i$, and $n$ is the number of nodes. |
| Characteristic path length[17] | The characteristic path length ($L$) measuring the whole brain integration | $L = \frac{1}{n}\sum_{i \in N} L_i = \frac{1}{n}\sum_{i \in N} \frac{\sum_{j \in N, j \neq i} d_{ij}}{n-1}$ <br> where $d_{ij}$ is a shortest path length (distance) between nodes $i$ and $j$, and $L_i$ is a characteristic path length of node $i$. |
| Global efficiency[18] | Global efficiency ( $E$ ) inferring the global information processing and transfer. | $E = \frac{1}{n}\sum_{i \in N} E_i = \frac{1}{n}\sum_{i \in N} \frac{\sum_{j \in N, j \neq i} d_{ij}^{-1}}{n-1}$ <br> where $E_i$ is the efficiency of node $i$. |
| Network small- | Small-world networks are highly clustered than random | $S = \frac{C / C_{rand}}{L / L_{rand}}$ |

| | | |
|---|---|---|
| worldness[19] | networks and approximately the same characteristic path length as random networks[17] | where $C$ and $C_{rand}$ are the clustering coefficients, and $L$ and $L_{rand}$ are the characteristic path lengths of the tested network and a related random network, respectively. Small-world networks often have $S > 1$. |

**Supplementary Table S3** Abbreviations for the cortical regions

| AAL number | Abgreviation | AAL Regions |
|---|---|---|
| 1 | PreCG.L | Left Precentral gyrus |
| 2 | PreCG.R | Right Precentral gyrus |
| 3 | SFGdor.L | Left Superior frontal gyrus, dorsolateral |
| 4 | SFGdor.R | Right Superior frontal gyrus, dorsolateral |
| 5 | ORBsup.L | Left Supeiror frontal gyrus, orbital part |
| 6 | ORBsup.R | Right Supeiror frontal gyrus, orbital part |
| 7 | MFG.L | Left Middle frontal gyrus |
| 8 | MFG.R | Right Middle frontal gyrus |
| 9 | ORBmid.L | Left Middle frontal gyrus orbital part |
| 10 | ORBmid.R | Right Middle frontal gyrus orbital part |
| 11 | IFGoperc.L | Left Inferior frontal gyrus, opercular part |
| 12 | IFGoperc.R | Right Inferior frontal gyrus, opercular part |
| 13 | IFGtriang.L | Left Inferior frontal gyrus, triangular part |
| 14 | IFGtriang.R | Right Inferior frontal gyrus, triangular part |
| 15 | ORBinf.L | Left Inferior frontal gyrus, orbital part |
| 16 | ORBinf.R | Right Inferior frontal gyrus, orbital part |
| 17 | ROL.L | Left Rolandic operculum |
| 18 | ROL.R | Right Rolandic operculum |
| 19 | SMA.L | Left Supplementary motor area |
| 20 | SMA.R | Right Supplementary motor area |
| 21 | OLF.L | Left Olfactory Cortex |
| 22 | OLF.R | Right Olfactory Cortex |
| 23 | SFGmed.L | Left Superior frontal gyrus, medial |
| 24 | SFGmed.R | Right Superior frontal gyrus, medial |
| 25 | ORBsupmed.L | Left Superior frontal gyrus, medial orbital |
| 26 | ORBsupmed.R | Right Superior frontal gyrus, medial orbital |
| 27 | REC.L | Left Gyrus Rectus |

| | | |
|---|---|---|
| 28 | REC.R | Right Gyrus Rectus |
| 29 | INS.L | Left Insula |
| 30 | INS.R | Right Insula |
| 31 | ACG.L | Left Anterior cingulate and paracingulate gyri |
| 32 | ACG.R | Right Anterior cingulate and paracingulate gyri |
| 33 | DCG.L | Left Median cingulate and paracingulate gyri |
| 34 | DCG.R | Right Median cingulate and paracingulate gyri |
| 35 | PCG.L | Left Posterior cingulate gyrus |
| 36 | PCG.R | Right Posterior cingulate gyrus |
| 39 | PHG.L | Left Parahippocampal gyrus |
| 40 | PHG.R | Right Parahippocampal gyrus |
| 43 | CAL.L | Left Calcarine fissure and surrounding cortex |
| 44 | CAL.R | Right Calcarine fissure and surrounding cortex |
| 45 | CUN.L | Left Cuneus |
| 46 | CUN.R | Right Cuneus |
| 47 | LING.L | Left Lingual gyrus |
| 48 | LING.R | Right Lingual gyrus |
| 49 | SOG.L | Left Superior occipital gyrus |
| 50 | SOG.R | Right Superior occipital gyrus |
| 51 | MOG.L | Left Middle occipital gyrus |
| 52 | MOG.R | Right Middle occipital gyrus |
| 53 | IOG.L | Left Inferior occipital gyrus |
| 54 | IOG.R | Right Inferior occipital gyrus |
| 55 | FFG.L | Left Fusiform gyrus |
| 56 | FFG.R | Right Fusiform gyrus |
| 57 | PoCG.L | Left Postcentral gyrus |
| 58 | PoCG.R | Right Postcentral gyrus |
| 59 | SPG.L | Left Superior parietal gyrus |
| 60 | SPG.R | Right Superior parietal gyrus |
| 61 | IPL.L | Left Inferior parietal |

| | | |
|---|---|---|
| 62 | IPL.R | Right Inferior parietal |
| 63 | SMG.L | Left Supramarginal gyrus |
| 64 | SMG.R | Right Supramarginal gyrus |
| 65 | ANG.L | Left Angular gyrus |
| 66 | ANG.R | Right Angular gyrus |
| 67 | PCUN.L | Left Precuneus |
| 68 | PCUN.R | Right Precuneus |
| 69 | PCL.L | Left Paracentral lobule |
| 70 | PCL.R | Right Paracentral lobule |
| 79 | HES.L | Left Heschl gyrus |
| 80 | HES.R | Right Heschl gyrus |
| 81 | STG.L | Left Superior temporal gyrus |
| 82 | STG.R | Right Superior temporal gyrus |
| 83 | TPOsup.L | Left Temporal pole: superior temporal gyrus |
| 84 | TPOsup.R | Right Temporal pole: superior temporal gyrus |
| 85 | MTG.L | Left Middle temporal gyrus |
| 86 | MTG.R | Right Middle temporal gyrus |
| 87 | TPOmid.L | Left Temporal pole: middle temporal gyrus |
| 88 | TPOmid.R | Right Temporal pole: middle temporal gyrus |
| 89 | ITG.L | Left Inferior temporal gyrus |
| 90 | ITG.R | Right Inferior temporal gyrus |

**Supplementary Table S4** The coincident hubs in each group from the principal data

| Subgroup | | $V_{TAV}$ | $V_{TA}$ | $V_{TV}$ | $V_{AV}$ | $V_{TO}$ | $V_{AO}$ | $V_{VO}$ |
|---|---|---|---|---|---|---|---|---|
| Male | $M_T$ | 0.188 | 0.353 | 0.167 | 0.000 | 0.455* | 0.000 | 0.048 |
| | $M_A$ | 0.091 | 0.333 | 0.040 | 0.313 | 0.050 | 0.389* | 0.038 |
| | $M_V$ | 0.333 | 0.077 | 0.444 | 0.235 | 0.000 | 0.000 | 0.588* |
| Female | $M_T$ | 0.238 | 0.400* | 0.125 | 0.143 | 0.353 | 0.190 | 0.040 |
| | $M_A$ | 0.136 | 0.250 | 0.083 | 0.353 | 0.048 | 0.600* | 0.087 |
| | $M_V$ | 0.238 | 0.000 | 0.350 | 0.200 | 0.000 | 0.042 | 0.444* |
| Young | $M_T$ | 0.238 | 0.389 | 0.227 | 0.043 | 0.529* | 0.077 | 0.130 |
| | $M_A$ | 0.182 | 0.190 | 0.038 | 0.263 | 0.040 | 0.474* | 0.083 |
| | $M_V$ | 0.389 | 0.000 | 0.444 | 0.211 | 0.042 | 0.000 | 0.786* |
| Old | $M_T$ | 0.273 | 0.333 | 0.091 | 0.045 | 0.600* | 0.080 | 0.000 |
| | $M_A$ | 0.174 | 0.278 | 0.000 | 0.375 | 0.150 | 0.625* | 0.045 |
| | $M_V$ | 0.261 | 0.000 | 0.316 | 0.143 | 0.042 | 0.077 | 0.563* |
| Dementia | $M_T$ | 0.043 | 0.294 | 0.313 | 0.048 | 0.714* | 0.042 | 0.087 |
| | $M_A$ | 0.042 | 0.150 | 0.048 | 0.211 | 0.042 | 0.529* | 0.040 |
| | $M_V$ | 0.174 | 0.000 | 0.143 | 0.389 | 0.080 | 0.037 | 0.647* |

The Jaccard index was calculated for the coincident hub regions between MCNs of $M_T$, $M_A$, and $M_V$ and subnetworks of each partition such as $V_{TAV}$, $V_{TA}$, $V_{TV}$, $V_{AV}$, $V_{TO}$, $V_{AO}$, $V_{VO}$. The hub regions of each network are marked in Supplementary Fig. S3. Asterisk indicates the highest Jaccard index between hub regions of MCNs and subnetworks of each partition.

**Supplementary Table S5** The coincident hubs in each group from the replication data set

| Subgroup | | $V_{TAV}$ | $V_{TA}$ | $V_{TV}$ | $V_{AV}$ | $V_{TO}$ | $V_{AO}$ | $V_{VO}$ |
|---|---|---|---|---|---|---|---|---|
| Total | $M_T$ | 0.091 | 0.250 | 0.300 | 0.000 | 0.474* | 0.040 | 0.000 |
| | $M_A$ | 0.050 | 0.294 | 0.000 | 0.500* | 0.042 | 0.353 | 0.048 |
| | $M_V$ | 0.353 | 0.000 | 0.190 | 0.176 | 0.000 | 0.042 | 0.500* |
| Male | $M_T$ | 0.235 | 0.211 | 0.333* | 0.000 | 0.316 | 0.043 | 0.000 |
| | $M_A$ | 0.000 | 0.263 | 0.000 | 0.375 | 0.083 | 0.471* | 0.087 |
| | $M_V$ | 0.278 | 0.042 | 0.238 | 0.150 | 0.080 | 0.040 | 0.733* |
| Female | $M_T$ | 0.136 | 0.368 | 0.167 | 0.000 | 0.526* | 0.077 | 0.000 |
| | $M_A$ | 0.313 | 0.158 | 0.043 | 0.118 | 0.000 | 0.600* | 0.038 |
| | $M_V$ | 0.278 | 0.043 | 0.300 | 0.235 | 0.000 | 0.083 | 0.611* |

The Jaccard index was calculated for the coincident hub regions between MCNs of $M_T$, $M_A$, and $M_V$ and subnetworks of each partition such as $V_{TAV}$, $V_{TA}$, $V_{TV}$, $V_{AV}$, $V_{TO}$, $V_{AO}$, $V_{VO}$. The hub regions of each network are marked in Supplementary Fig. S6. Asterisk indicates the highest Jaccard index between hub regions of MCNs and subnetworks of each partition.

**Supplementary Fig. S1** Group differences in ORs from the principal data

The plot figure shows the observed OR of each partition as a function. Each plot was marked as follows: (a) Male group, (b) Female group, (c) Group difference in OR between male and female group, (d) Old group, (e) Young group, (f) Group difference in OR between old and young group, (g) dementia group, (h) Group difference in OR between old and dementia group. In (c), (f), and (h), data points with cross indicate statistically significance ($P < 0.05$).

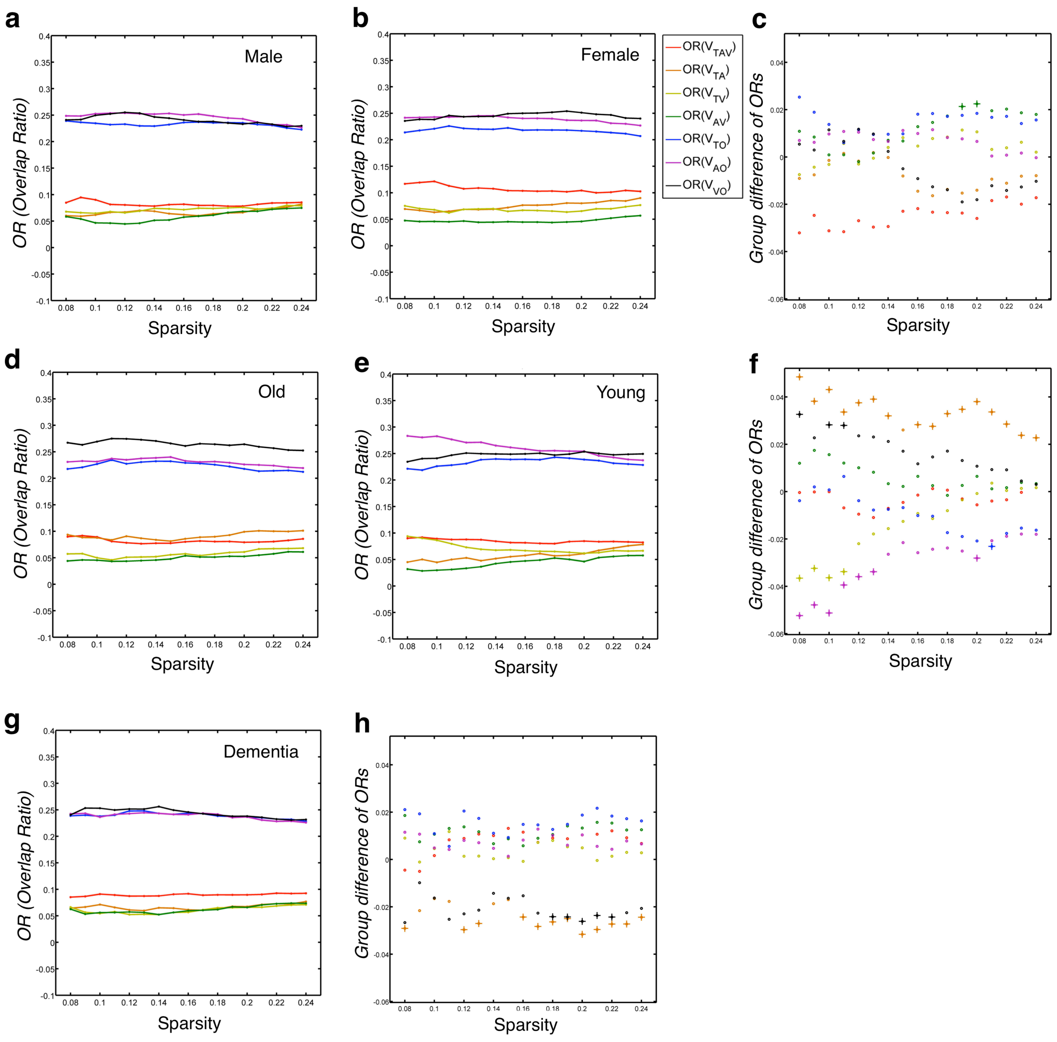

**Supplementary Fig. S2** Four edge types for subgroups from the principal data

(a) The edges show both signs, with positive and negative correlation coefficients between regions. (b) The edges show only positive correlation. (c) The edges show only negative correlation. The percentages of the four types of edges, that is, the homotopic, the left and right ipsilateral, and heterotopic edges were plotted in each partition. In each box, the central mark is the median, the edges of the box are the 25th and 75th percentiles, and the box color indicates the edge type. The box plot was implemented using matlab code (alex.bikfalvi.com/research/advanced_matlab_boxplot/).

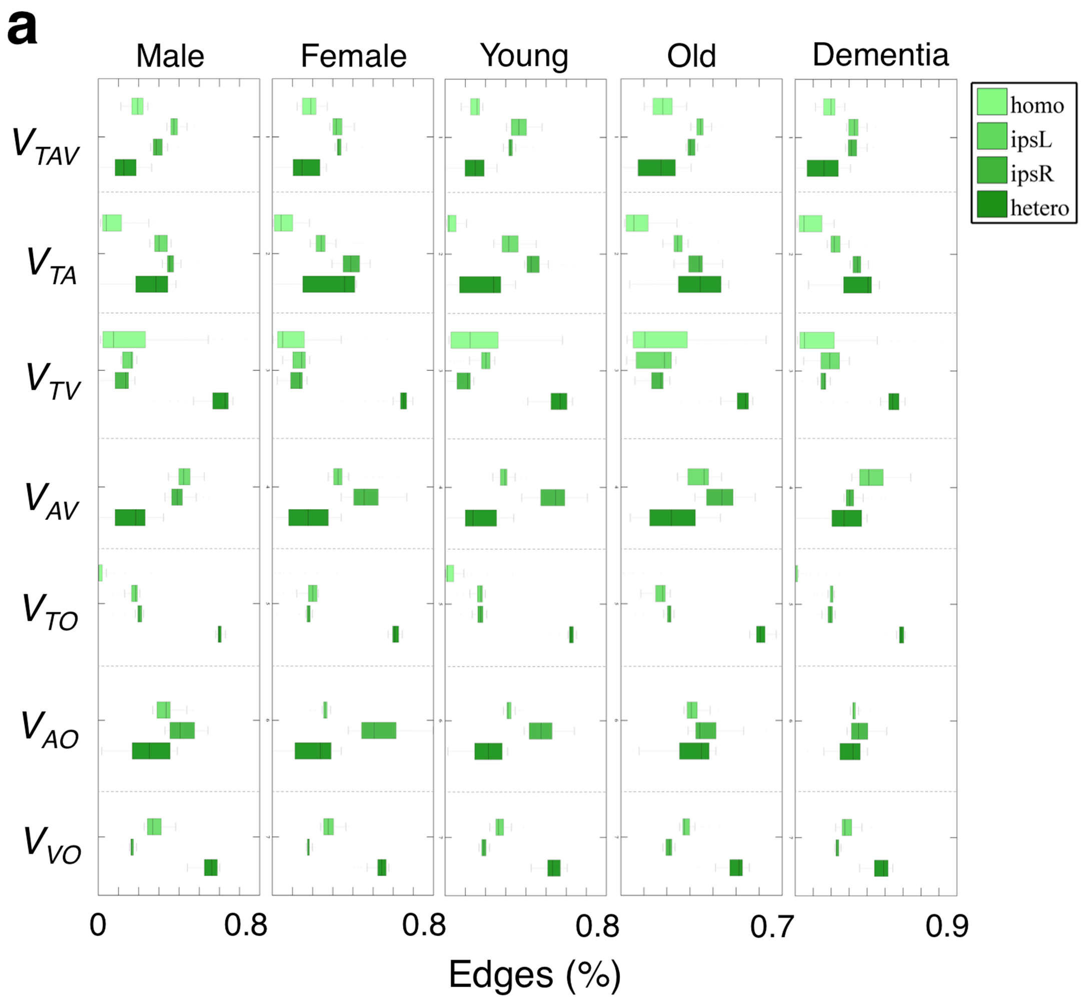

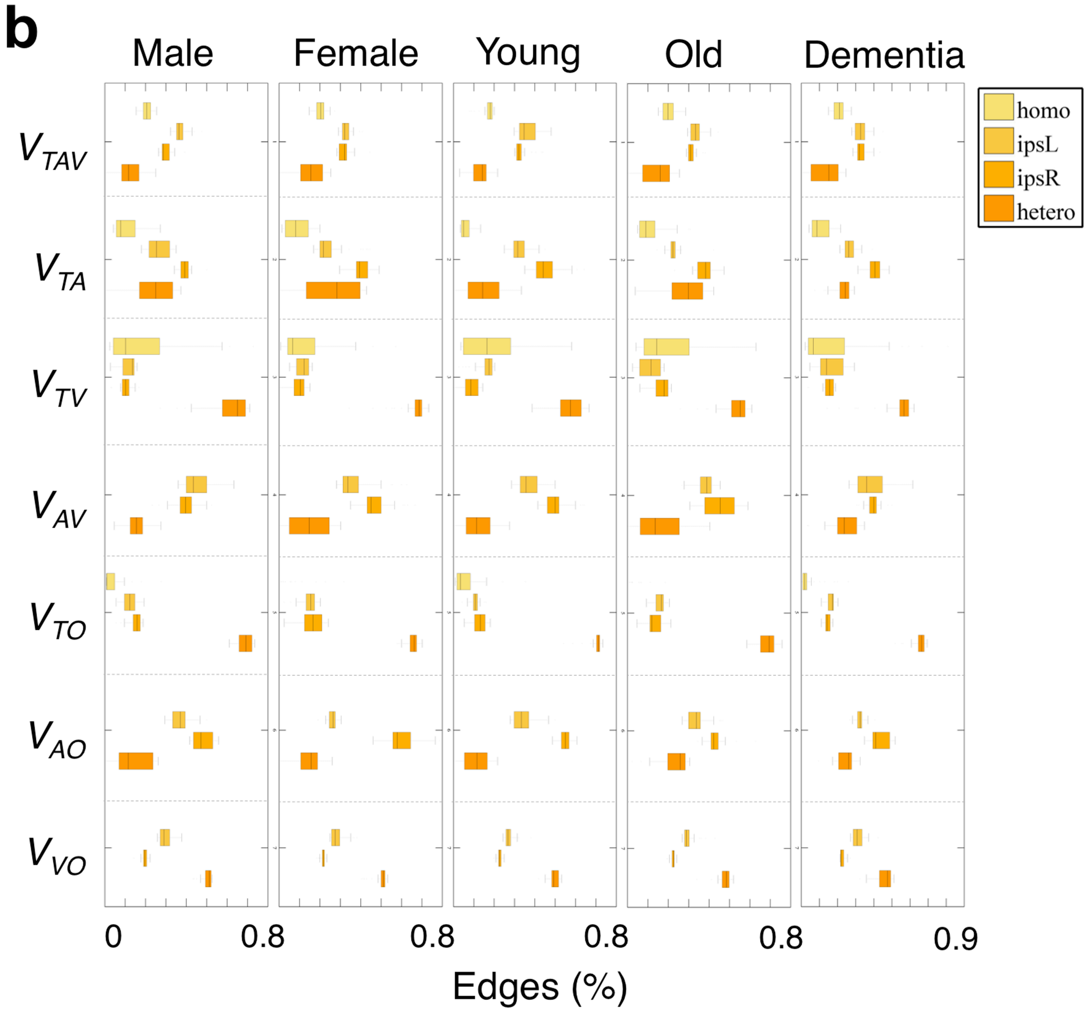

b
Male
Female
Young
Old
Dementia
homo
ipsL
ipsR
hetero
$V_{TAV}$
$V_{TA}$
$V_{TV}$
$V_{AV}$
$V_{TO}$
$V_{AO}$
$V_{VO}$
0
0.8
0.8
0.8
0.8
0.9
Edges (%)

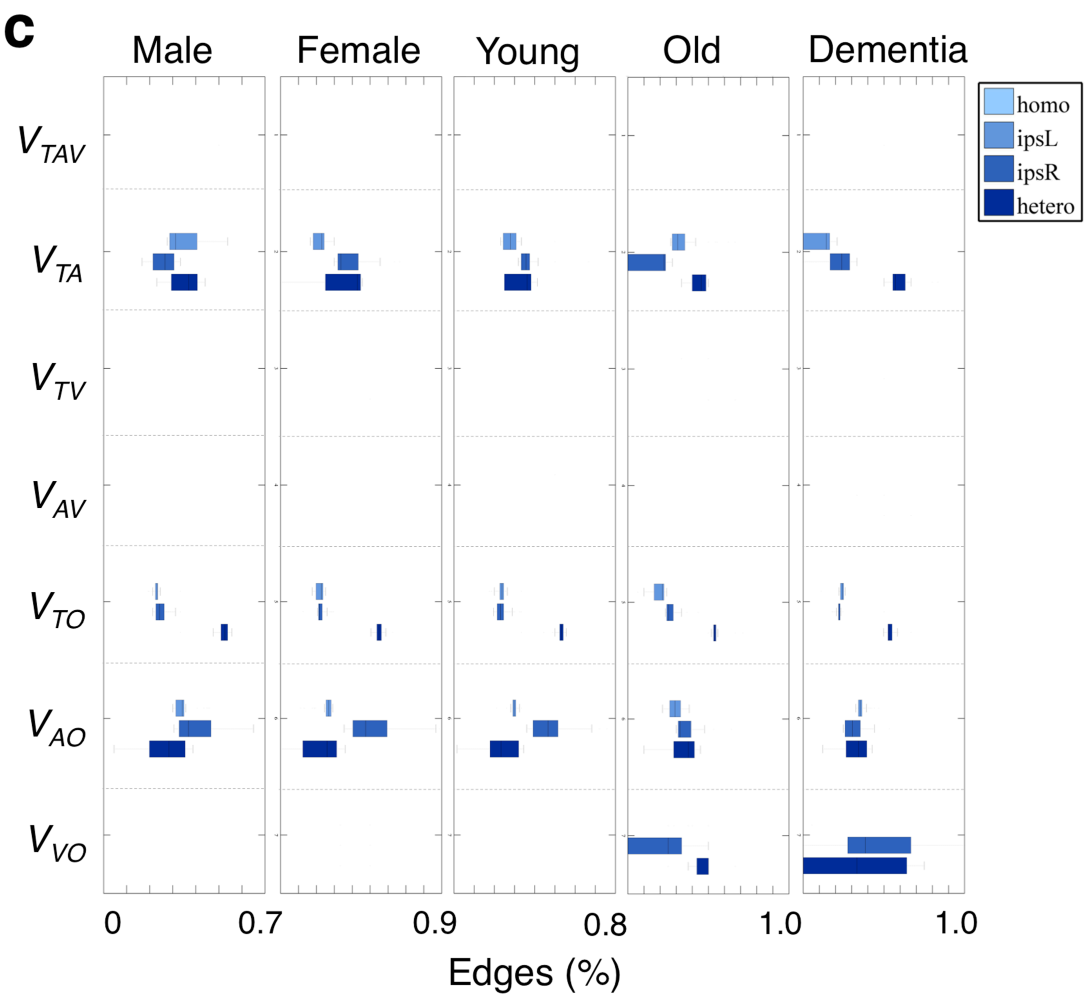
c
Male
Female
Young
Old
Dementia
homo
ipsL
ipsR
hetero
$V_{TAV}$
$V_{TA}$
$V_{TV}$
$V_{AV}$
$V_{TO}$
$V_{AO}$
$V_{VO}$
0
0.7
0.9
0.8
1.0
1.0
Edges (%)

**Supplementary Fig. S3** The hub regions from the principal data

Each partition (a-j) was marked as follows: (a) $V_{TAV}$, (b) $V_{TA}$, (c) $V_{TV}$, (d) $V_{AV}$, (e) $V_{TO}$, (f) $V_{AO}$, (g) $V_{VO}$, and (h) $M_T$, (i) $M_A$, and (j) $M_V$ indicate the adjacency matrix of cortical thickness, surface area, and gray matter volume, respectively. The hub regions for each group were marked as different markers with same color in each box plot. In each box, each group was marked as follows: M; Male group, F; Female group, Y; Young group, O; Old, D; Dementia group.

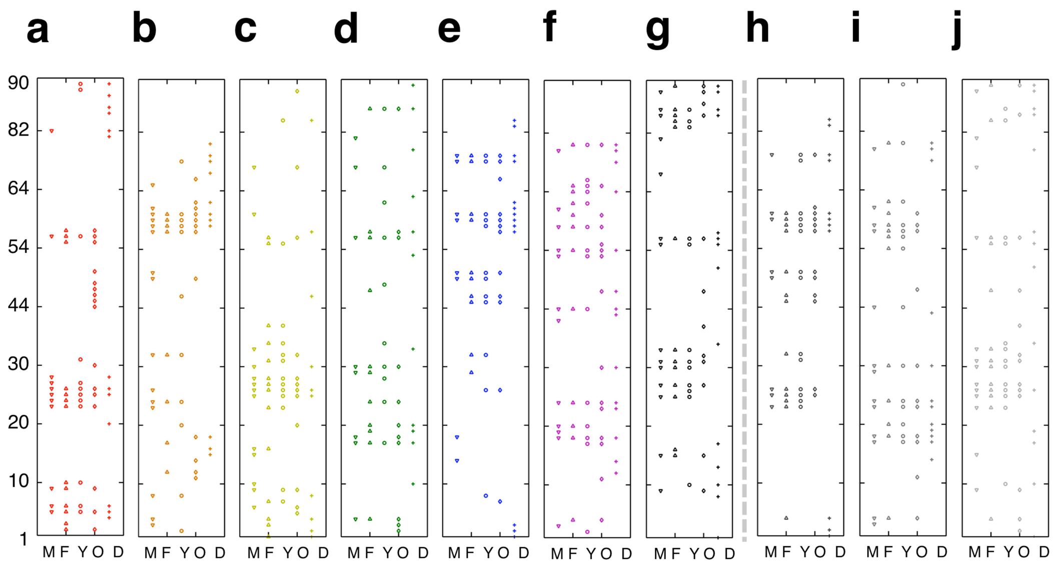

**Supplementary Fig. S4** Group differences in ORs from the replication data

The plot figure shows the observed OR of each partition as a function.

Each plot was marked as follows: (a) Total subjects, (b) Male group, (c) Female group, (d) Group difference in OR between male and female group and data points with cross indicate statistically significance ($P < 0.05$).

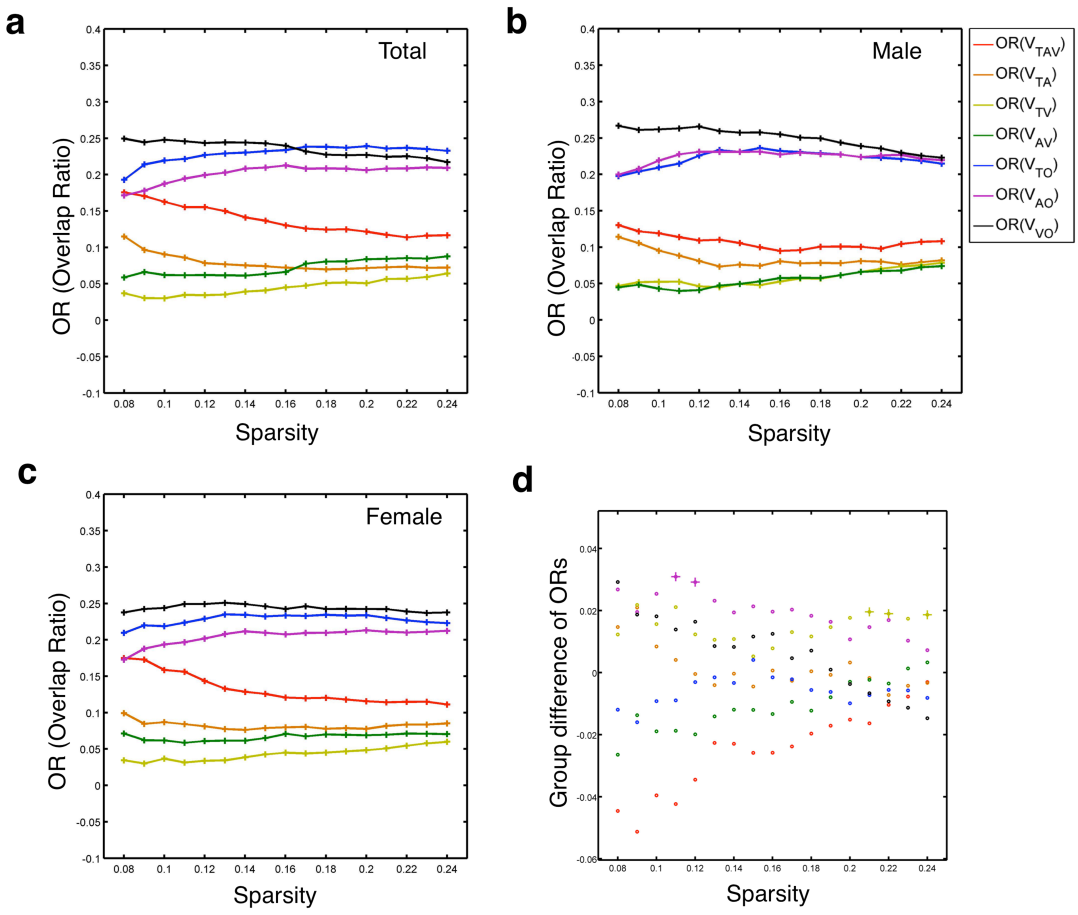

**Supplementary Fig. S5** Four edge types for subgroups from the replication data

The percentages of the four types of edges, that is, the homotopic, the left and right ipsilateral, and heterotopic edges were plotted in each partition. Each group was marked as follows: (a) total subjects, (b) male group, (c) female group. Green box plots indicate the edges showing both signs, with positive and negative correlation coefficients between regions. Orange box plots indicate the edges showing only positive correlation. Blue box plots indicate the edges showing only negative correlation. In each box, the central mark is the median, the edges of the box are the 25th and 75th percentiles, and the box color indicates the edge type.

**a**

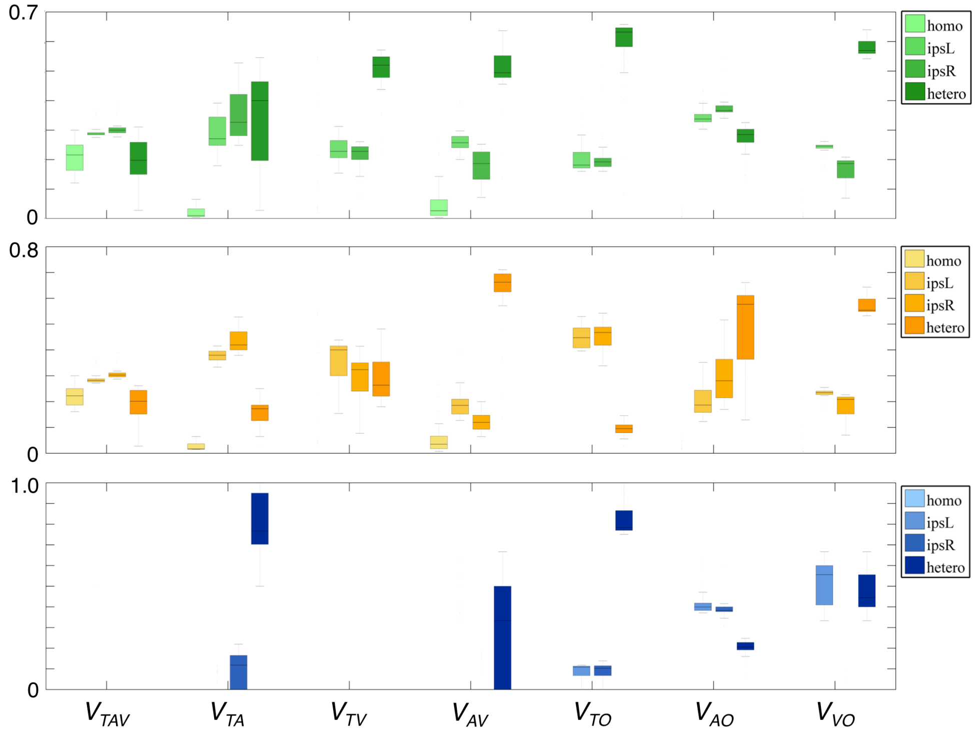

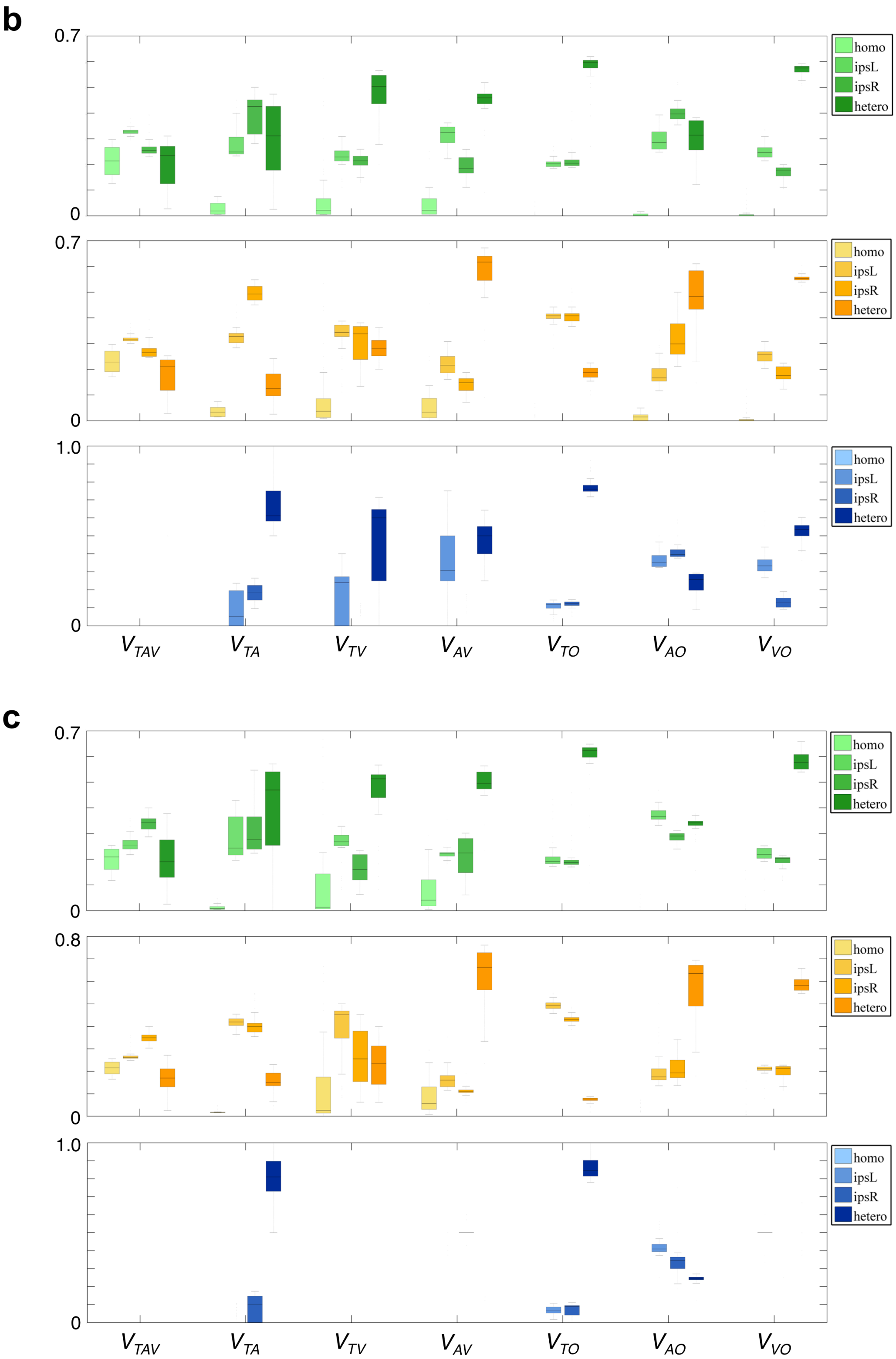

b
0.7
0
homo
ipsL
ipsR
hetero
0.7
0
1.0
0
$V_{TAV}$
$V_{TA}$
$V_{TV}$
$V_{AV}$
$V_{TO}$
$V_{AO}$
$V_{VO}$
c
0.7
0
0.8
0
1.0
0

**Supplementary Fig. S6** The hub regions from the replication data

Each partition (a-j) was marked as follows: (a) $V_{TAV}$, (b) $V_{TA}$, (c) $V_{TV}$, (d) $V_{AV}$, (e) $V_{TO}$, (f) $V_{AO}$, (g) $V_{VO}$, and (h) $M_T$, (i) $M_A$, and (j) $M_V$ indicate the adjacency matrix of cortical thickness, surface area, and gray matter volume, respectively. The hub regions for each group were marked as different markers with same color in each box plot. In each box, each group was marked as follows: Total; total subjects, M; Male group, F; Female group.

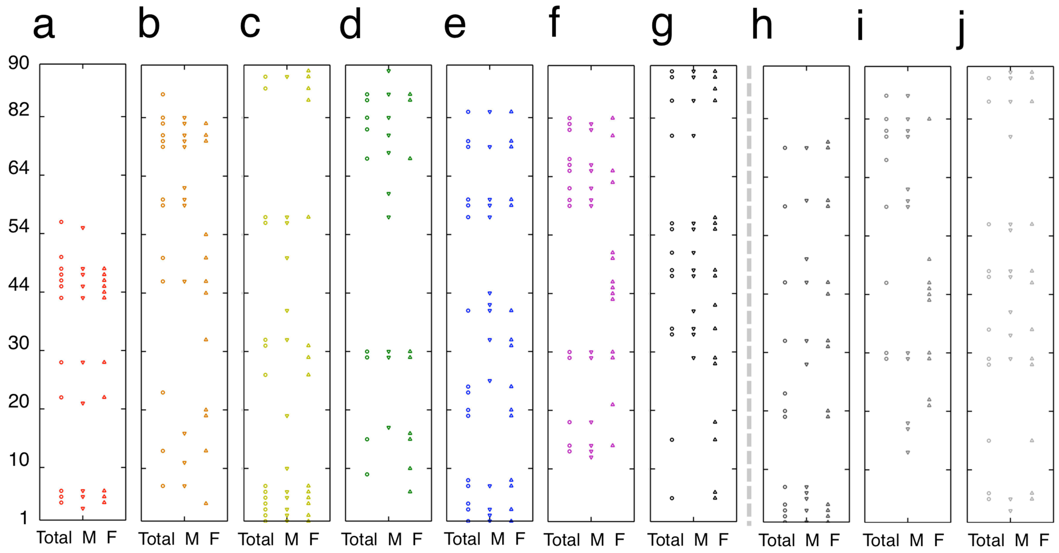

**Supplementary Fig. S7** Comparison of network properties between three networks. Network properties of (a) small-worldness index, (b) normalized clustering coefficients, (c) global efficiency, and (d) normalized characteristic path length as functions of network sparsity for three MCNs of cortical thickness (red), surface area (blue), and gray matter volume (black). The network parameters of 1000 bootstrap samples show the mean and standard deviation at each sparsity level. Data points with asterisks indicate significant differences ($P < 0.05$).

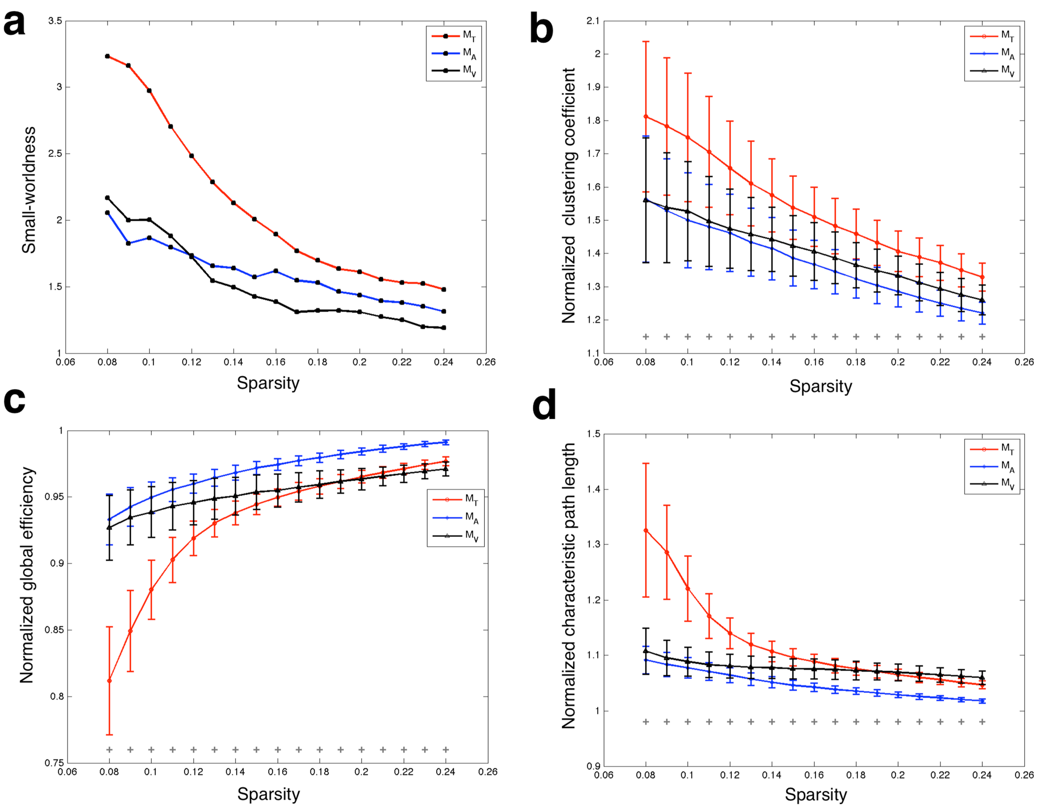

## Supplementary Data

**Principal data set** The OASIS data (www.oasis-brains.org) were composed of 416 subjects with ages from 18–96 years including 316 of healthy controls and 100 of patients with Dementia on 1.5 Tesla MRI. For this study, 2 of normal subjects and 11 of patients were excluded because of failure of image processing. We selected 314 healthy normal subjects, referred to as the principal data set of the study. The subjects were 44.9 ± 23.79 years old (mean ± standard deviation (STD)) with a sex ratio of 195/119 (female/male). For group differences, we grouped the subjects as follows: (1) Female and male group; their ages were ranged 47.9 ± 24.39 and 39.9 ± 22.0 respectively for the female and male groups. (2) Young and old group; 119 subjects of 20s (22.8 ± 2.49 years old, 68/51 (female/male)) were selected as young group and with old group, 96 subjects (75.8 ± 8.99 years old, 70/26 (female/male)), aged 60 and older in the principal data. (3) For patient group, 89 of dementia subjects (76.8 ± 7.25 years old, 55/34 (female/male)) were included in this study.

All the magnetic resonance imaging (MRI) data were sagittal T1-weighted images with typical dimensions of 256 × 256 and resolutions of 1 mm × 1 mm × 1.25 mm. The scans were collected using a Siemens Vision scanner with a magnetization-prepared rapid acquisition with gradient echo. All subjects used in this work gave written informed consent and the use of these subjects was approved by the Institutional Review Boards (IRB) of Washington University (www.oasis-brains.org). The details have been described in Marcus, D. S. et al (2007) for OASIS[20].

**Replication data set** An independent replication data set was obtained from HCP data (www.humanconnectome.org) including 509 healthy subjects with age range of 22 to 35 years (302/207 (female/male)). The data set were provided by the Human

Connectome Project, WU-Minn Consortium (Principal Investigators: David Van Essen and Kamil Ugurbil; 1U54MH091657) funded by the 16 NIH Institutes and Centers that support the NIH Blueprint for Neuroscience Research; and by the McDonnell Center for Systems Neuroscience at Washington University.

All images were scanned on a customized Siemens 3 T (a modified Siemens Skyra 3T) using the special gradients of the WU-Minn and MGH-UCLA Connectome scanners. Briefly, T1-weighted images are acquired using the 3D MPRAGE sequence with 0.7 mm isotropic resolution and the following parameters: TR=2400ms, TE=2.14ms, TI=1000ms, FA=8°, Band- width (BW) = 210 Hz per pixel, Echo Spacing (ES) = 7.6 ms, with a non-selective binomial (1:1) water excitation pulse (a pair of 100 µs hard pulses with 1.2 ms spacing). The field of view (FOV) was 224 mm, matrix size was 320 x 256 with sagittal slices in a single slab. The details have been described in Van Essen, D. C. et al. for HCP[21,22].

# Supplementary Methods

## Image preprocessing for a morphometric descriptor

CIVET software was used to extract cortical surfaces. The procedures are well validated and have been extensively described[23-27]. The T1-weighted MRI data were first registered into a standardized stereotaxic space using an affine linear transformation and then corrected for nonuniform intensity artifacts using an N3 algorithm[28,29]. The registered and corrected volumes were segmented into GM, white matter (WM), cerebrospinal fluid (CSF), and background using a neural net classifier[30]. Next, the inner and outer surfaces were automatically constructed by deforming a spherical mesh onto the GM/WM boundary and GM/CSF boundary, respectively, in each hemisphere using the CLASP algorithm[31,32]. A surface model for each hemisphere consisted of 81924 vertices with polygonal meshes. We applied an inverse transformation matrix to the surface model to estimate cortical measurements in the native space. Cortical thickness was calculated as the Euclidean distance between the corresponding vertices of inner and outer surfaces using a t-link method[23]. Surface area was measured by calculating the Voronoi area assigned to any vertices[33]. Cortical thickness and surface area were blurred using a 20 mm full-width half-maximum surface-based diffusion kernel and spatially normalized to a group template using a surface-based two-dimensional registration algorithm[34,35]. GM volume was obtained from the segmented GM images in native space.

## Morphometric network construction

For each subject, regional cortical thickness and surface area were respectively obtained as the average thickness of all the vertices belonging to each region and the sum of the surface area making up the surface model within each region[33,36]. The

regional GM volume was calculated by measuring the total number of voxels belonging to that region. A linear regression analysis was performed at every cortical region to remove the confounding factors of age, sex, and any variance explained by global measures (mean cortical thickness, and total sum of surface area, estimated intracranial volume, respectively)[4,8,9,15] The interregional symmetric correlation matrix was constructed by calculating the Pearson correlation coefficients across the individuals between the morphological measures of every regional pair.

## References


1 He, Y., Chen, Z. J. & Evans, A. C. Small-world anatomical networks in the human brain revealed by cortical thickness from MRI. *Cereb Cortex* **17**, 2407-2419, doi:DOI 10.1093/cercor/bhl149 (2007).

2 Bassett, D. S. *et al.* Hierarchical organization of human cortical networks in health and schizophrenia. *The Journal of neuroscience : the official journal of the Society for Neuroscience* **28**, 9239-9248, doi:10.1523/JNEUROSCI.1929-08.2008 (2008).

3 Chen, Z. J., He, Y., Rosa, P., Germann, J. & Evans, A. C. Revealing modular architecture of human brain structural networks by using cortical thickness from MRI. *Cereb Cortex* **18**, 2374-2381, doi:DOI 10.1093/cercor/bhn003 (2008).

4 He, Y., Chen, Z. & Evans, A. Structural insights into aberrant topological patterns of large-scale cortical networks in Alzheimer's Disease. *Journal of Neuroscience* **28**, 4756-4766, doi:Doi 10.1523/Jneurosci.0141-08.2008 (2008).

5 He, Y. *et al.* Impaired small-world efficiency in structural cortical networks in multiple sclerosis associated with white matter lesion load. *Brain* **132**, 3366-3379, doi:Doi 10.1093/Brain/Awp089 (2009).

6 Lv, B. *et al.* Gender consistency and difference in healthy adults revealed by cortical thickness. *Neuroimage* **53**, 373-382, doi:10.1016/j.neuroimage.2010.05.020 (2010).

7 Raj, A., Mueller, S. G., Young, K., Laxer, K. D. & Weiner, M. Network-level analysis of cortical thickness of the epileptic brain. *Neuroimage* **52**, 1302-1313, doi:10.1016/j.neuroimage.2010.05.045 (2010).

8 Sanabria-Diaz, G. *et al.* Surface area and cortical thickness descriptors reveal different attributes of the structural human brain networks. *Neuroimage* **50**, 1497-1510, doi:DOI 10.1016/j.neuroimage.2010.01.028 (2010).

9 Bernhardt, B. C., Chen, Z., He, Y., Evans, A. C. & Bernasconi, N. Graph-Theoretical Analysis Reveals Disrupted Small-World Organization of Cortical Thickness Correlation Networks in Temporal Lobe Epilepsy. *Cereb Cortex* **21**, 2147-2157, doi:DOI 10.1093/cercor/bhq291 (2011).

10 Fan, Y. *et al.* Brain anatomical networks in early human brain development. *Neuroimage* **54**, 1862-1871, doi:10.1016/j.neuroimage.2010.07.025 (2011).

11 Zhou, L., Wang, Y., Li, Y., Yap, P. T. & Shen, D. Hierarchical anatomical brain networks for MCI prediction: revisiting volumetric measures. *PloS one* **6**, e21935, doi:10.1371/journal.pone.0021935 (2011).

12 Hosseini, S. M., Koovakkattu, D. & Kesler, S. R. Altered small-world properties of gray matter networks in breast cancer. *BMC neurology* **12**, 28, doi:10.1186/1471-2377-12-28 (2012).

13 Shi, F. *et al.* Altered structural connectivity in neonates at genetic risk for schizophrenia: a combined study using morphological and white matter networks. *Neuroimage* **62**, 1622-1633, doi:10.1016/j.neuroimage.2012.05.026 (2012).

14 Wu, K. *et al.* Age-related changes in topological organization of structural brain networks in healthy individuals. *Human brain mapping* **33**, 552-568, doi:10.1002/hbm.21232 (2012).

15 Hosseini, S. M. *et al.* Topological properties of large-scale structural brain

networks in children with familial risk for reading difficulties. *Neuroimage* **71**, 260-274, doi:10.1016/j.neuroimage.2013.01.013 (2013).
16 Rubinov, M. & Sporns, O. Complex network measures of brain connectivity: uses and interpretations. *Neuroimage* **52**, 1059-1069, doi:10.1016/j.neuroimage.2009.10.003 (2010).
17 Watts, D. J. & Strogatz, S. H. Collective dynamics of 'small-world' networks. *Nature* **393**, 440-442, doi:Doi 10.1038/30918 (1998).
18 Latora, V. & Marchiori, M. Efficient behavior of small-world networks. *Physical review letters* **87**, 198701 (2001).
19 Humphries, M. D. & Gurney, K. Network 'Small-World-Ness': A Quantitative Method for Determining Canonical Network Equivalence. *PloS one* **3**, doi:ARTN e0002051
10.1371/journal.pone.0002051 (2008).
20 Marcus, D. S. *et al.* Open Access Series of Imaging Studies (OASIS): cross-sectional MRI data in young, middle aged, nondemented, and demented older adults. *J Cogn Neurosci* **19**, 1498-1507, doi:10.1162/jocn.2007.19.9.1498 (2007).
21 Van Essen, D. C. *et al.* The WU-Minn Human Connectome Project: an overview. *Neuroimage* **80**, 62-79, doi:10.1016/j.neuroimage.2013.05.041 (2013).
22 Glasser, M. F. *et al.* The minimal preprocessing pipelines for the Human Connectome Project. *Neuroimage* **80**, 105-124, doi:10.1016/j.neuroimage.2013.04.127 (2013).
23 Lerch, J. P. & Evans, A. C. Cortical thickness analysis examined through power analysis and a population simulation. *Neuroimage* **24**, 163-173, doi:DOI 10.1016/j.neuroimage.2004.07.045 (2005).
24 Kabani, N., Le Goualher, G., MacDonald, D. & Evans, A. C. Measurement of cortical thickness using an automated 3-D algorithm: A validation study. *Neuroimage* **13**, 375-380, doi:DOI 10.1006/nimg.2000.0652 (2001).
25 Lerch, J. P. *et al.* Focal decline of cortical thickness in Alzheimer's disease identified by computational neuroanatomy. *Cereb Cortex* **15**, 995-1001, doi:DOI 10.1093/cercor/bhh200 (2005).
26 Lee, J. K. *et al.* A novel quantitative cross-validation of different cortical surface reconstruction algorithms using MRI phantom. *Neuroimage* **31**, 572-584, doi:10.1016/j.neuroimage.2005.12.044 (2006).
27 Singh, V. *et al.* Spatial patterns of cortical thinning in mild cognitive impairment and Alzheimer's disease. *Brain* **129**, 2885-2893, doi:Doi 10.1093/Brain/Awl256 (2006).
28 Sled, J. G., Zijdenbos, A. P. & Evans, A. C. A nonparametric method for automatic correction of intensity nonuniformity in MRI data. *IEEE transactions on medical imaging* **17**, 87-97, doi:10.1109/42.668698 (1998).
29 Collins, D. L., Neelin, P., Peters, T. M. & Evans, A. C. Automatic 3D intersubject registration of MR volumetric data in standardized Talairach space. *Journal of computer assisted tomography* **18**, 192-205 (1994).
30 Zijdenbos, A. *et al.* Automatic quantification of multiple sclerosis lesion volume using stereotaxic space. *Lect Notes Comput Sc* **1131**, 439-448 (1996).
31 Kim, J. S. *et al.* Automated 3-D extraction and evaluation of the inner and outer cortical surfaces using a Laplacian map and partial volume effect classification. *Neuroimage* **27**, 210-221, doi:DOI 10.1016/j.neuroimage.2005.03.036 (2005).

32 MacDonald, D., Kabani, N., Avis, D. & Evans, A. C. Automated 3-D extraction of inner and outer surfaces of cerebral cortex from MRI. *Neuroimage* **12**, 340-356, doi:10.1006/nimg.1999.0534 (2000).

33 Im, K. *et al.* Brain size and cortical structure in the adult human brain. *Cereb Cortex* **18**, 2181-2191, doi:10.1093/cercor/bhm244 (2008).

34 Robbins, S., Evans, A. C., Collins, D. L. & Whitesides, S. Tuning and comparing spatial normalization methods. *Medical image analysis* **8**, 311-323, doi:DOI 10.1016/j.media.2004.06.009 (2004).

35 Lyttelton, O., Boucher, M., Robbins, S. & Evans, A. An unbiased iterative group registration template for cortical surface analysis. *Neuroimage* **34**, 1535-1544, doi:DOI 10.1016/j.neuroimage.2006.10.041 (2007).

36 Im, K. *et al.* Fractal dimension in human cortical surface: multiple regression analysis with cortical thickness, sulcal depth, and folding area. *Human brain mapping* **27**, 994-1003, doi:10.1002/hbm.20238 (2006).
